# New Staphylinidae (Coleoptera) records with new collection data from New Brunswick, Canada: Paederinae

**DOI:** 10.3897/zookeys.186.2504

**Published:** 2012-04-26

**Authors:** Reginald P. Webster, Ian DeMerchant

**Affiliations:** 1 Natural Resources Canada, Canadian Forest Service - Atlantic Forestry Centre, 1350 Regent St., P.O. Box 4000, Fredericton, NB, Canada E3B 5P7

**Keywords:** Paederinae, new records, Canada, New Brunswick

## Abstract

We report 17 species of Paederinae new for New Brunswick, Canada. Ten of these species, *Lathrobium othioides* LeConte, *Lathrobium amplipenne* Casey, *Lathrobium armatum* Say, *Lathrobium confusum* LeConte, *Lathrobium debile* LeConte, *Achenomorphus corticinus* (Gravenhorst), *Rugilus rufipes* Germar, *Homaeotarsus bicolor* (Gravenhorst), *Homaeotharsus cribratus* (LeConte), and *Homaeotarsus pallipes* (Gravenhorst) are newly recorded for the Maritime provinces. This brings the total number of Paederinae recorded from New Brunswick to 36 species. Additional records are presented for the recently reported *Lathrobium simile* LeConte and *Lathrobium washingtoni* Casey. Collection and habitat data are presented for all species.

## Introduction

This paper treats new Staphylinidae records from New Brunswick of the subfamily Paederinae. The most recent taxonomic treatments of the North American Paederinae fauna were by Casey (1905, 1910). More recently Watrous (1981) reviewed the *Tetartopeus* (as a subgenus of *Lathrobium*) and the subgenus *Eulathrobium* of *Lobrathium*. Herman (1965a, b) revised the genus *Orus*. Many other genera in North America are in need of a modern revision.

Many species of Paederinae in Canada occur in and near wetland habitats such as marshes, bogs, and pond and river margins (Watrous 1980, 1981; Newton et al. 2000; Brunke et al. 2011). A few species, such as *Sunius confluentus* (Say), occur in subcortical habitats (Brunke et al. 2011). However, relatively little has been published on the bionomics of species occurring in Canada or North America.

Campbell and Davies (1991) reported 15 species of Paederinae from New Brunswick. Klimaszewski et al. (2005) added *Lathrobium simile* LeConte, *Lathrobium washingtoni* Casey, and *Ochthephilum fracticorne* (Paykull) from their study on rove beetles in red spruce (*Picea rubens* Sarg.) stands. The adventive *Lathrobium fulvipenne* (Gravenhorst) was reported from New Brunswick by Majka and Klimaszewski (2008). Here, we report an additional 17 species of Paederinae for New Brunswick, bringing the total number of species known from the province to 36.

## Methods and conventions

The following records are based on specimens collected as part of a general survey to document the Coleoptera fauna of New Brunswick and from by-catch samples from Lindgren 12-funnel traps (Lindgren 1983) obtained during a study to develop a general attractant for the detection of invasive species of Cerambycidae.

### Collection methods

Various collection methods were employed to collect the species reported in this study. Details are outlined in Campbell (1973) and Webster et al. (2009, Appendix). See Webster et al. (2012) for details of the methods used for deployment of Lindgren traps and sample collection. A description of the habitat was recorded for all specimens collected during this survey. Locality and habitat data are presented exactly as recorded on labels for each specimen. This information, as well as additional collecting notes, is summarized and discussed in the collection and habitat data section for each species.

### Specimen preparation

Males and some females of most species were dissected to confirm their identity. The genital structures were dehydrated in absolute alcohol, mounted in Canada balsam on celluloid microslides, and pinned with the specimens from which they originated.

### Distribution

Distribution maps, created using ArcMap and ArcGIS, are presented for each species in New Brunswick. Every species is cited with current distribution in Canada and Alaska, using abbreviations for the state, provinces, and territories. New provincial records are indicated in bold under Distribution in Canada and Alaska. The following abbreviations are used in the text.

Acronyms of collections examined and referred to in this study are as follows:

**Table:** 

**AK**	Alaska	**MB**	Manitoba
**YT**	Yukon Territory	**ON**	Ontario
**NT**	Northwest Territories	**QC**	Quebec
**NU**	Nunavut	**NB**	New Brunswick
**BC**	British Columbia	**PE**	Prince Edward Island
**AB**	Alberta	**NS**	Nova Scotia
**SK**	Saskatchewan	**NF & LB**	Newfoundland and Labrador*

*Newfoundland and Labrador are each treated separately under the current Distribution in Canada and Alaska.

**AFC** Atlantic Forestry Centre, Natural Resources Canada, Canadian Forest Service, Fredericton, New Brunswick, Canada

**CNC** Canadian National Collection of Insects, Arachnids and Nematodes, Agriculture and Agri-Food Canada, Ottawa, Ontario, Canada

**NBM** New Brunswick Museum, Saint John, New Brunswick, Canada

**RWC** Reginald P. Webster Collection, Charters Settlement, New Brunswick, Canada

## Results

Unless noted otherwise (additional records), all records below are species newly recorded for New Brunswick, Canada. Species followed by ** are newly recorded from the Maritime provinces (New Brunswick, Nova Scotia, Prince Edward Island) of Canada.

## Species accounts

The classification of the Paederinae follows Bouchard et al. (2011).

Seventeen species of Paederinae are newly reported for the New Brunswick, Canada. Ten of these are newly recorded for the Maritime provinces. This brings the total number of Paederinae recorded from New Brunswick to 36 (Table 1). Additional records and bionomic data of the recently reported *Lathrobium simile* and *Lathrobium washingtoni* are presented.

**Table 1.** Species of Paederinae (Staphylinidae) recorded from New Brunswick, Canada.

**Subfamily Paederinae Fleming**
**Tribe Paederini Fleming**
**Subtribe Lathrobiina Laporte**
*Lathrobium (Lathrobioma) scolopaceum* (Casey)
*Lathrobium (Lathrobioma) othioides* LeConte**
*Lathrobium (Lathrobium) amplipenne* Casey**
*Lathrobium (Lathrobium) armatum* Say**
*Lathrobium (Lathrobium) confusum* LeConte**
*Lathrobium (Lathrobium) fauveli* Duvivier
*Lathrobium (Lathrobium) fulvipenne* (Gravenhorst)
*Lathrobium (Lathrobium) simile* LeConte
*Lathrobium (Lathrobium) sparsellum* Casey
*Lathrobium (Lathrobium) spissicorne* Casey*
*Lathrobium (Lathrobium) washingtoni* Casey
*Lathrobium (Lathrolepta) debile* LeConte**
*Lobrathium (Lobrathium) collare* (Erichson)*
*Lobrathium (Eulathrobium) grande* (LeConte)*
*Tetartopeus angularis* (LeConte)
*Tetartopeus furvulus* Casey
*Tetartopeus lacustris* Casey
*Tetartopeus niger* (LeConte)
*Tetartopeus nitidulus* (LeConte)
*Tetartopeus capitosus* Casey*
*Tetartopeus rubripennis* Casey*
**Subtribe Medonina Casey**
*Achenomorphus corticinus* (Gravenhorst)**
*Lithocaris (Lithocharis) ochracea* (Gravenhorst)
*Pseudomedon thoracica* Casey
*Sunius (Trachysectus) confluentus* (Say)
**Subtribe Stilicina Casey**
*Pachystilicus hanhami* (Wickham)
*Rugilus angustatus* (Geoffrey)*
*Rugilus biarmatus* (LeConte)
*Rugilus rufipes* Germar**
**Subtribe Astenina Hatch**
*Astenus discopunctatus* (Say)
**Subtribe Cryptobiina Casey**
*Homaeotarsus (Gastrolobium) bicolor* (Gravenhorst)**
*Homaeotarsus (Hesperobium) cinctus* (Say)*
*Homaeotarsus (Hesperobium) cribratus* (LeConte)**
*Homaeotarsus (Hesperobium) pallipes* (Gravenhorst)**
*Ochthephilum fracticorne* (Paykull)
**Subtribe Paederina Fleming**
*Paederus littorarius* (Gravenhorst)

**Notes:** *New to province, **New to Maritime Provinces

### Family Staphylinidae Latreille, 1802

**Subfamily Paederinae Fleming, 1821**

**Tribe Paederini Fleming, 1821**

**Subtribe Lathrobiina Laporte, 1835**

#### Lathrobium (Lathrobioma) othioides

LeConte, 1880**

http://species-id.net/wiki/Lathrobium_othioides

Map 1

##### Material examined.

**New Brunswick, Carleton Co.**, Richmond, Hovey Hill Protected (Natural) Area, 46.1115°N, 67.7770°W, 10.V.2005,R. P. Webster, hardwood forest, in moist leaf litter and moss near seepage area (3 ♂, RWC). **Sunbury Co.**, Acadia Research Forest, 45.9816°N, 66.3374°W, 18.VI.2007, R. P. Webster, 8.5 year-old regenerating mixed forest, sifting leaf litter (1 ♂, AFC). **York Co.**, Charters Settlement, 45.8428°N, 66.7279°W, 19.IV.2004, 24.IV.2004, 15.IV.2005, 20.IV.2005, R. P. Webster, mixed forest, sedge marsh in moist grass litter and sphagnum (6 ♂, RWC).

**Map 1.**
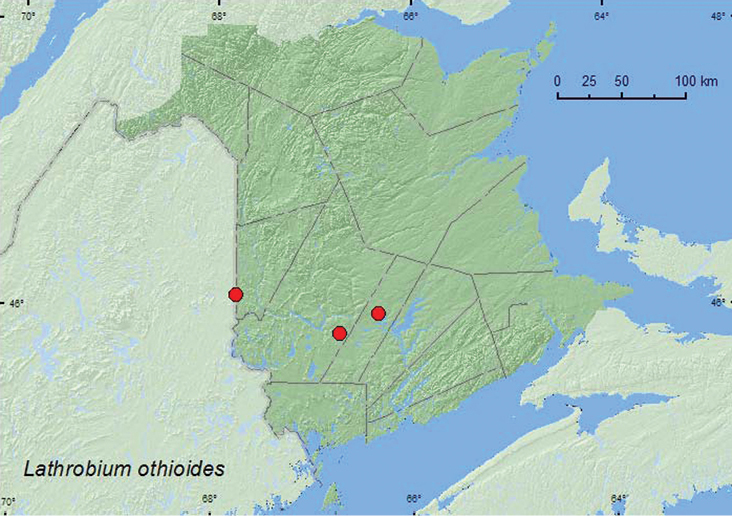
Collection localities in New Brunswick, Canada of *Lathrobium othioides*.

##### Collection and habitat data.

*Lathrobium othioides* was found in an old hardwood forest, a regenerating mixed forest, and a sedge (*Carex*) marsh. Adults were sifted from leaf litter, moist leaf litter, and moss in a seepage area and from moist grass litter and sphagnum. This species was collected during April, May, and June.

##### Distribution in Canada and Alaska.

ON, QC, **NB** (Campbell and Davies 1991).

#### Lathrobium (Lathrobium) amplipenne

Casey, 1905**

http://species-id.net/wiki/Lathrobium_amplipenne

Map 2

##### Material examined.

**New Brunswick, Restigouche Co.**, Little Tobique River near Red Brook, 47.4465°N, 67.0689°W, 13.VI.2006, R. P. Webster, river margin in *Carex* hummock (1 ♂, 1 ♀, RWC). **Sunbury Co.**, Sheffield, Portobello Creek N.W.A., 45.8952°N, 66.2728°W, 7.V.2004, R. P. Webster, silver maple swamp, in leaf litter (1 ♀, RWC).

**Map 2.**
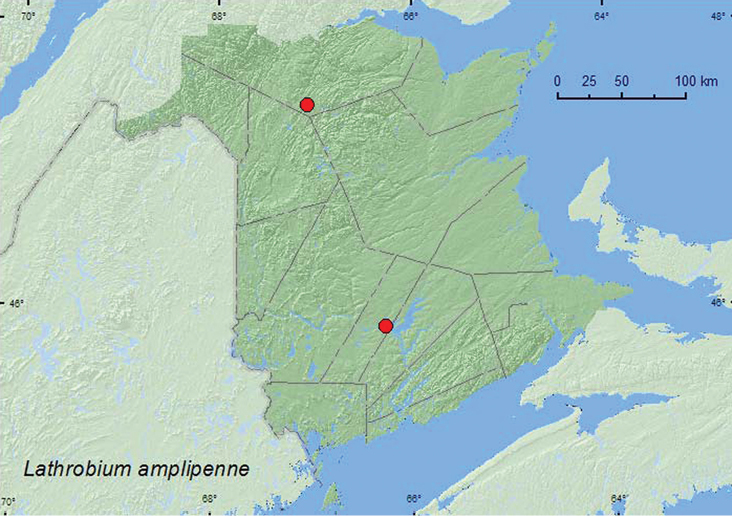
Collection localities in New Brunswick, Canada of *Lathrobium amplipenne*.

##### Collection and habitat data.

Adults were sifted from a *Carex* hummock on a river margin and from leaf litter in a silver maple (*Acer saccharinum* L.) swamp. Adults were collected during May and June.

##### Distribution in Canada and Alaska.

ON, **NB** (Campbell and Davies 1991).

#### Lathrobium (Lathrobium) armatum

Say, 1834**

http://species-id.net/wiki/Lathrobium_armatum

Map 3

##### Material examined.

**New Brunswick, Carleton Co.** Jackson Falls, Bell Forest, 46.2150°N, 67.7201°W, 14.V.2006, R. P. Webster, river margin, in drift material near seepage area (1 ♂, 1 ♀, RWC). **York Co.**, Dumfries, Slagundy Dry Ponds, 45.8596°N, 67.1849°W, 8.VII.2006, R. P. Webster, large vernal pond, pond margin in moist leaves (1 ♂, 2 ♀, RWC); 8.5 km W of Tracy off Rt. 645, 45.6888°N, 66.8004°W, 22.V.2008, R. P. Webster, *Carex* marsh/flowage near slow flowing brook, in *Carex* hummocks (3 ♂, 1 ♀, RWC).

**Map 3:**
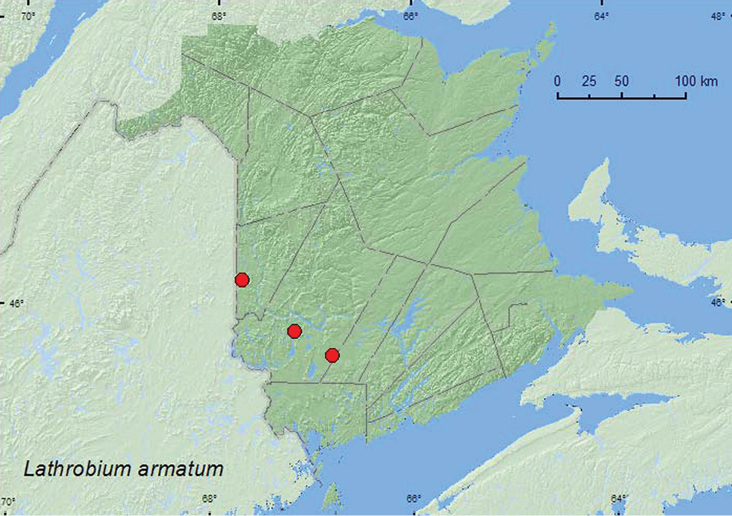
**.** Collection localities in New Brunswick, Canada of *Lathrobium armatum*.

##### Collection and habitat data.

*Lathrobium armatum* was sifted from drift material near a seepage area along a river margin, from *Carex* hummocks in a *Carex* marsh/flowage near a slow-flowing brook and from moist leaves on the margin of a large vernal pond. Adults were collected during May and July.

##### Distribution in Canada and Alaska.

ON, QC, **NB** (Campbell and Davies 1991).

#### Lathrobium (Lathrobium) confusum

LeConte, 1880**

http://species-id.net/wiki/Lathrobium_confusum

Map 4

##### Material examined.

**New Brunswick, Carleton Co.**, near Hovey Hill P.N.A., 46.1155°N, 67.7631°W, 10.V.2005, R. P. Webster, mixed forest, in (leaf) litter near small brook (1 ♂, RWC); Meduxnekeag Valley Nature Preserve, 46.1964°N, 67.6840°W, 31.V.2005, M.-A. Giguère & R. Webster, mixed forest, vernal pond margin in moist (leaf) litter (2 ♀, NBM, RWC); same locality and forest type but, 46.1976°N, 67.6850°W, margin of vernal pond, in moist leaves (1 ♂, NBM). **Queens Co.**, W of Jemseg at “Trout Creek”, 45.8227°N, 66.1240°W, 26.IV.2004, 9.V.2004, R. P. Webster, silver maple swamp, sifting (leaf) litter at bases of large trees (2 ♂, 1 ♀, RWC); same locality, forest type, and collector but 45.8231°N, 66.1245°W, 11.IV.2006, sifting litter from crotch of silver maple with multiple trunks (1 ♂, NBM). **Sunbury Co.**, Sheffield, Portobello Creek N.W.A., 45.8952°N, 66.2728°W, 7.V.2004, R. P. Webster, silver maple swamp, in leaf litter (1 ♀, RWC); Burton, Sunpoke Lake, 45.7575°N, 66.5736°W, 30.IV.2004, 10.IV.2006, R. P. Webster, red maple swamp, in leaf litter near slow stream (1 ♂, 3 ♀, NBM, RWC). **York Co.** Kelly’s Creek at Sears Rd., 45.8723°N, 66.8414°W, 7.VI.2008, R.P. Webster, alder swamp with red maples, in moist leaf and grass litter near (small) pool (1 ♂, RWC).

**Map 4.**
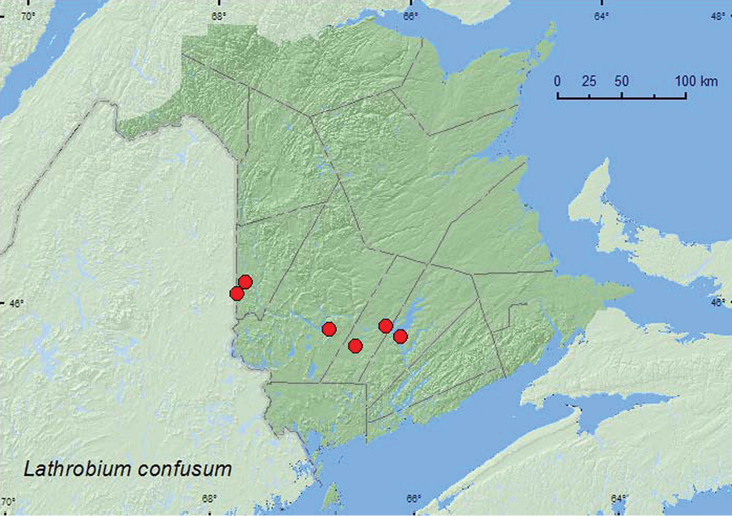
Collection localities in New Brunswick, Canada of *Lathrobium confusum*.

##### Collection and habitat data.

Watrous (1980) reported *Lathrobium confusum* from the margin of an intermittent stream. In New Brunswick, adults were found in leaf litter or grass litter near small brooks and streams and in leaf litter along vernal pond and forest pool margins. Some were sifted from leaf litter at the base of large silver maples or from litter in the crotch of a silver maple with multiple trunks. Adults were found in mixed forests, silver maple swamps, red maple (*Acer rubrum* L.) swamps, and an alder (*Alnus* sp.) swamp with red maple. This species was collected during April, May, and June.

##### Distribution in Canada and Alaska.

ON, QC, **NB** (Campbell and Davies 1991).

#### Lathrobium (Lathrobium) simile

LeConte, 1863

http://species-id.net/wiki/Lathrobium_simile

Map 5

##### Material examined.

**Additional New Brunswick records, Carleton Co.**, Hovey Hill P.N.A., 46.1115°N, 67.7770°W, 10.V.2005, R. P. Webster, hardwood forest, in moist leaf litter and moss near forest pool (1 ♂, RWC); Meduxnekeag Valley Nature Preserve, 46.1956°N, 67.6803°W, 15.IX.2004, R. P. Webster, mixed forest, in decaying fungi (1 ♂, RWC); same locality, forest type and collector but 46.1976°N, 67.6850°W, 4.V.2006, R. P. Webster, margin of vernal pond, in moist leaf litter (1 ♂, RWC); Jackson Falls, Bell Forest, 46.2210°N, 67.7210°W, 11.V.2005, M.-A. Giguère & R. Webster, hardwood forest, in leaf litter near small brook (1 ♂, 2 ♀, RWC). **Sunbury Co.**, Acadia Research Forest, 30.VI.1999, G. Gesner, Strip Cut 8, Site 1, pitfall trap (1, AFC); Acadia Research Forest, 45.9799°N, 66.3394°W, 18.VI.2007, R. P. Webster, mature red spruce and red maple forest, sifting leaf litter (1 ♂, 1 ♀, RWC).

**Map 5.**
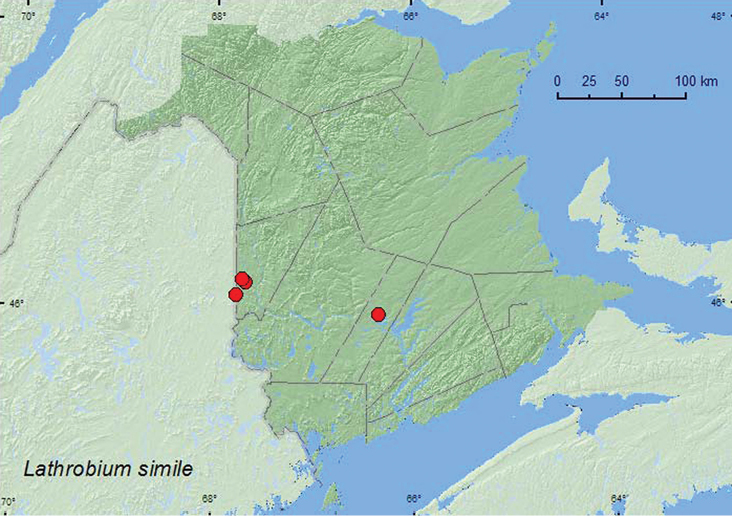
Collection localities in New Brunswick, Canada of *Lathrobium simile*.

##### Collection and habitat data. 

Adults of this species were found in hardwood forests, mixed forests, and a mature red spruce forest. Adults were collected from moist leaf litter near forest pools or vernal ponds, near a small brook, and from the forest floor. One individual was collected from decaying fungi on the forest floor. This species was collected during May, June, and September.

##### Distribution in Canada and Alaska.

MB, ON, QC, NB, NS (Campbell and Davies 1991; Klimaszewski et al. 2005). This species was first reported from New Brunswick by Klimaszewski et al. (2005) from the Acadia Research Forest.

#### Lathrobium (Lathrobium) spissicorne

Casey, 1905

http://species-id.net/wiki/Lathrobium_spissicorne

Map 6

##### Material examined.

**New Brunswick, Charlotte Co.**, near New River, 45.2118°N, 66.6179°W, 7.VII.2006, R. P. Webster, mixed forest, margin small pond, treading *Carex* hummock into water (1 ♀, RWC). **Queens Co.**, W of Jemseg at “Trout Creek”, 45.8240°N, 66.1220°W, 4.VI.2004, R. P. Webster, silver maple swamp, margin of vernal pond in moist leaf litter on muddy soil (1 ♀, RWC); Grand Lake near Scotchtown, 45.8762°N, 66.1816°W, 5.VI.2004, 1.VII.2004, 25.V.2006, R. P. Webster, lakeshore, old (sand) dune with oaks, under dead fish and under drift material (3 ♂, 1 ♀, NBM, RWC). **Sunbury Co.**, Burton, Sunpoke Lake, 45.7665°N, 66.5545°W, 15.V.2004, R. P. Webster, (red) oak and (red and silver) maple forest, in leaf litter (3 ♂, RWC).

**Map 6.**
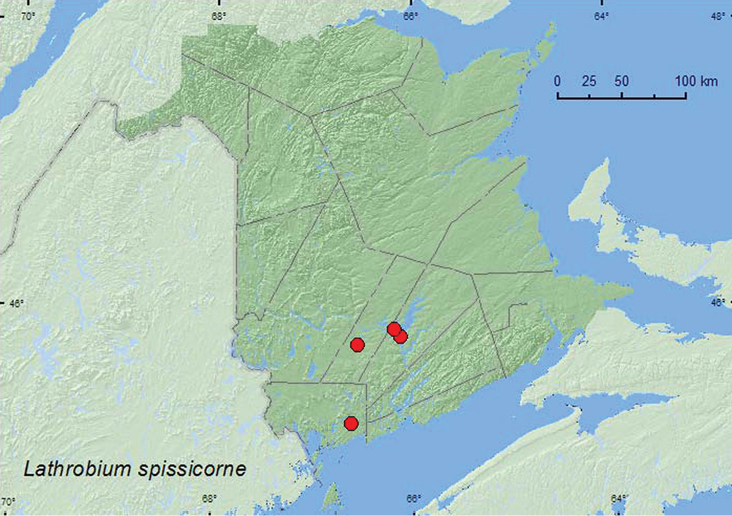
Collection localities in New Brunswick, Canada of *Lathrobium spissicorne*.

##### Collection and habitat data.

*Lathrobium spissicorne* was found in mixed forests, a silver maple swamp, along a lakeshore, and in a mature forest with red oak (*Quercus rubra* L.), red maple, and silver maple. Adults were found in a *Carex* hummock on a pond margin, in moist leaf litter on vernal pond margin, and in leaf litter on the forest floor. Some adults were found under drift material and under a dead fish on a lakeshore. This species was collected during May, June, and July.

##### Distribution in Canada and Alaska.

ON, QC, **NB**, PE (Campbell and Davies 1991).

#### Lathrobium (Lathrobium) washingtoni

Casey, 1905

http://species-id.net/wiki/Lathrobium_washingtoni

Map 7

##### Material examined.

**Additional New Brunswick records, Queens Co.**, W of Jemseg at “Trout Creek”, 45.8227°N, 66.1240°W, 4.VI.2004, R. P. Webster, silver maple swamp, sifting litter at base of large tree (silver maple) (1 ♂, RWC). **Saint John Co.**, ca. 2 km NE of Maces Bay, 45.1168°N, 66.4552°W, 8.V.2006, R. P. Webster, eastern white cedar swamp, in sphagnum and leaf litter (1 ♀, RWC). **Sunbury Co.**, Burton, SW of Sunpoke Lake, 45.7575°N, 66.5726°W, 17.IV.2005, R. P. Webster, red maple swamp, in leaf litter near margin of slow stream (1 ♂, RWC); Acadia Research Forest, 45.9799°N, 66.3394°W, 14.V.2007, R. P. Webster, mature red spruce and red maple forest, sifting leaf litter (1 ♂, AFC). **York Co.**, Charters Settlement, 45.8331°N, 66.7410°W, 16.IV.2004, R. P. Webster, mature red spruce and eastern white cedar forest, in moss and litter near small brook (1 ♂, RWC); same locality, forest type, and collector but 45.8341°N, 66.7445°W, 27.IV.2005, margin of vernal pond in leaf litter (1 ♂, NBM); New Maryland, off Hwy 2, E of Baker Brook, 45.8760°N, 66.6252°W, 6.IV.2005, 26.IV.2005, 4.VI.2005, R. P. Webster, old growth eastern white cedar swamp, in moss and litter at base of cedar (3 ♂, NBM, RWC); Canterbury, Browns Mountain Fen, 45.8967°N, 67.6343°W, 1.VI.2005, M.-A. Giguère and R. Webster, calcareous fen, in moist sphagnum (1 ♂, NBM); Mazerolle Settlement, 45.8729°N, 66.8311°W, 28.IV.2006, R. P. Webster, eastern white cedar swamp, margin of vernal pool in leaf litter (1 ♂, 1 ♀, RWC).

**Map 7.**
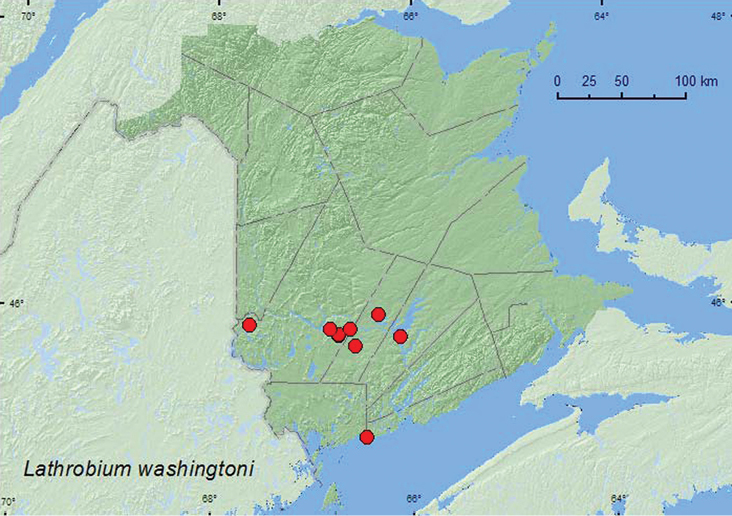
Collection localities in New Brunswick, Canada of *Lathrobium washingtoni*.

##### Collection and habitat data.

*Lathrobium washingtoni* was found in eastern white cedar (*Thuja occidentalis* L.) swamps, a silver maple swamp, a red maple swamp, a mature red spruce forest, and in an open calcareous cedar fen. Adults were sifted from sphagnum, sphagnum and leaf litter, moss and/or litter at bases of trees (silver maple, eastern white cedar), leaf litter near the margin of a slow stream and a brook, and from leaf litter on vernal pond margins. Adults were collected during April, May, and June.

##### Distribution in Canada and Alaska.

AK, NT, BC, AB, SK, MB, ON, QC, NB, NS, NF (Campbell and Davies 1991; Klimaszewski et al. 2005). This species was first reported from New Brunswick by Klimaszewski et al. (2005) from the Acadia Research Forest.

#### Lathrobium (Lathrolepta) debile

LeConte, 1880**

http://species-id.net/wiki/Lathrobium_debile

Map 8

##### Material examined.

**New Brunswick, Charlotte Co.**, at New River, 45.2166°N, 66.5953°W, 2.VI.2006, R. P. Webster, river margin, under debris (1 ♂, RWC). **Queens Co.**, W of Jemseg at “Trout Creek”, 45.8227°N, 66.1240°W, 4.VI.2004, 3.IV.2006, 11.IV.2006, R. P. Webster, silver maple swamp, sifting litter at base of large tree (silver maple) (2 ♂, 1 ♀, NBM, RWC); Grand Lake near Scotchtown, 45.8762°N, 66.1816°W, 25.IV.2004, 12.V.2004, R. P. Webster, (red) oak and (silver) maple forest, in leaf litter (2 ♂, 2 ♀, RWC). **Sunbury Co.**, Burton, SW of Sunpoke Lake, 45.7575°N, 66.5726°W, 17.IV.2005, R. P. Webster, red maple swamp, in leaf litter near margin of slow stream (1 ♀, NBM). **York Co.**, Charters Settlement, 45.8380°N, 66.7310°W, 18.IV.2004, R. P. Webster, mixed forest, in leaf litter near stream (1 ♀, RWC); 8.5 km W of Tracy off Rt. 645, 45.6821°N, 66.7894°W, 6.V.2008, R. P. Webster, alder swamp, in leaf litter and grass on hummocks (1, RWC); Fredericton, Odell Park, 45.9570°N, 66.6695°W, 19.VI.2005, R. P. Webster, old growth hemlock forest, in leaf litter (1 ♂, RWC).

**Map 8.**
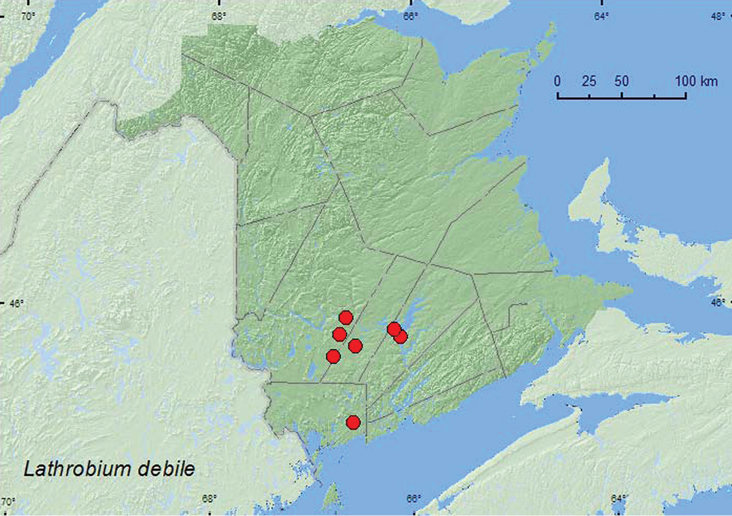
Collection localities in New Brunswick, Canada of *Lathrobium debile*.

##### Collection and habitat data.

In New Brunswick, *Lathrobium debile* was found in a silver maple swamp, a red oak and silver maple forest, a red maple swamp, a mixed forest, an alder swamp, an old-growth eastern hemlock (*Tsuga canadensis* (L.) Carr.) forest, and along a river margin. Most adults were sifted from leaf litter on the forest floor or near stream margins. One individual was found under drift material on a river margin. Adults were collected during April, May, and June.

##### Distribution in Canada and Alaska.

ON, QC, **NB** (Campbell and Davies 1991).

#### Tetartopeus capitosus

Casey, 1905

http://species-id.net/wiki/Tetartopeus_capitosus

Map 9

##### Material examined.

**New Brunswick, Queens Co.**, W of Jemseg at “Trout Creek”, 45.8240°N, 66.1220°W, 4.VI.2004, R. P. Webster, silver maple swamp, margin of vernal pond in moist leaf litter on muddy soil (1 ♀, RWC); Grand Lake near Scotchtown, 45.8762°N, 66.1816°W, R. P. Webster, 5.VI.2004, lake margin, under drift material (1 ♀, RWC); same locality data and collector, 25.V.2006, oak and maple forest near lakeshore, in litter near vernal pond (1 ♂, 1 ♀, RWC); Upper Gagetown, bog adjacent to Hwy 2, 45.8316°N, 66.2346°W, 23.V.2006, R. P. Webster, tamarack bog, treading *Carex* into water (1 ♂, RWC). **Sunbury Co.**, Maugerville, Portobello Creek N.W.A., 45.8992°N, 66.4248°W, 5.VI.2004, R. P. Webster, silver maple swamp, margin of small (vernal) pond, in leaf litter (1 ♀, RWC); Sheffield, Portobello Creek N.W.A., 45.8952°N, 66.2728°W, 17.VII.2004, R. P. Webster, silver maple swamp, u.v. light (1 ♀, RWC).

**Map 9.**
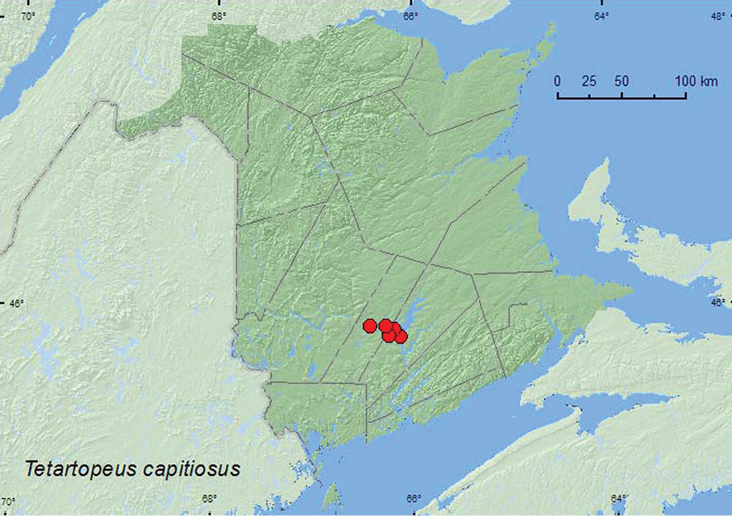
Collection localities in New Brunswick, Canada of *Tetartopeus capitosus*.

##### Collection and habitat data.

Nearly all *Tetartopeus* spp. have been collected in association with wetland habitats, usually in accumulations of damp leaf litter, moss, and other debris along streams, bogs, marshes, swamps, and ponds (Watrous 1980). Watrous (1980) noted that *Tetartopeus capitosus* (as *Latrobium (Tetartopeus) punctulatum* LeConte) occurred mostly in the boreal forest region but gave no specific habitat data for this species. In New Brunswick, adults of this species were found in silver maple swamps (floodplain forests), a red oak and silver maple forest, along a lake margin, and in a tamarack (*Larix laricina* (Du Roi) K. Koch) bog. Adults were found in leaf litter near vernal ponds, under drift material on a lake margin, and by treading *Carex* in a tamarack bog, and one individual was captured at an ultraviolet light.

##### Distribution in Canada and Alaska.

AK, NT, BC, AB, SK, MB, ON, QC, **NB**, NS (Watrous 1980; Campbell and Davies 1991).

#### Tetartopeus rubripennis

Casey, 1905

http://species-id.net/wiki/Tetartopeus_rubripennis

Map 10

##### Material examined.

**New Brunswick, Madawaska Co.**, at Green River, 47.6918°N, 68.3202°W, 21.VI.2010, M. Turgeon & R. Webster, river margin among gravel on gravel bar (1 ♀, RWC). **Restigouche Co.**, Jacquet River Gorge P.N.A., 47.8197°N, 66.0835°W, 26.VI.2008, R. P. Webster, margin of Jacquet River among cobblestones near water (1 ♂, RWC); same locality and collector but 47.7894°N, 66.1065°W, 14.V.2010, river margin (Jacquet River), under drift material (1 ♂, RWC); same locality and collector but 47.8257°N, 66.0779°W, 24.V.2010, partially shaded cobblestone bar near outflow of brook at Jacquet River, under cobblestones and gravel (1 ♀, RWC); Kedgwick Forks, 47.9085°N, 67.9057°W, 22.VI.2010, R. P. Webster, river margin, gravel bar among gravel and cobblestones (1 ♂, 2 ♀, RWC).

**Map 10.**
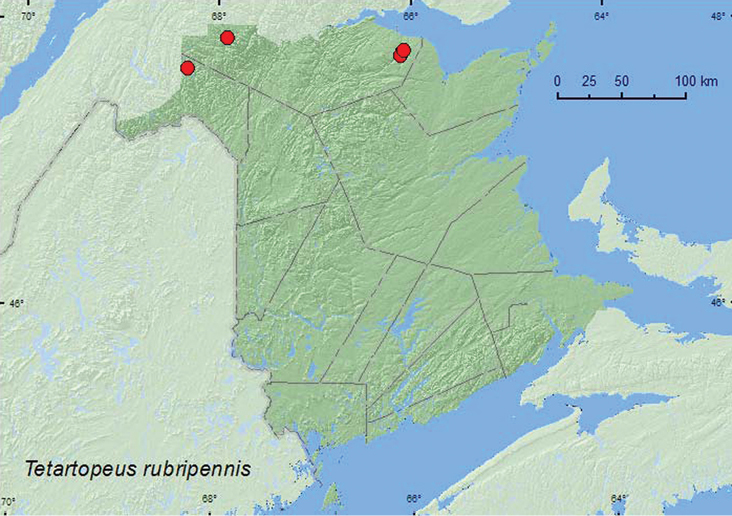
Collection localities in New Brunswick, Canada of *Tetartopeus rubripennis*.

##### Collection and habitat data.

Watrous (1980) reported *Tetartopeus rubripennis* (as *Lathrobium (Tetartopeus) rubripenne*) primarily from thin litter in marshes at one locality. In northern New Brunswick, *Tetartopeus rubripennis* was found along river margins among gravel and cobblestones or under drift material, usually near water. Adults were collected during May and June.

##### Distribution in Canada and Alaska.

ON, QC, **NB**, NS (Campbell and Davies 1991). In the description of *Tetartopeus rubripennis*, Watrous (1980) noted that the elytra are usually entirely red and rarely black with only apices reddish. Only one of the seven specimens of this species from New Brunswick had entirely reddish elytra. However, the male genitalia of the dark specimens conform to the illustration (Fig. 51) given in Watrous (1980) for *Tetartopeus rubripennis*. There are also specimens of the dark form from Nova Scotia and Quebec in the CNC (A. Davies, personal communication).

#### Lobrathium (Eulathrobium) grande

(LeConte, 1863)

http://species-id.net/wiki/Lobrathium_grande

Map 11

##### Material examined.

**New Brunswick, Charlotte Co.**, near Clark Ridge, 45.3155°N, 67.4406°W, 27.V.2007, R. P. Webster, beaver pond, treading vegetation (1, NBM). **Queens Co.**, Scotchtown near Indian Point (at Grand Lake), 45.8762°N, 66.1816°W, R. P. Webster, 5.VI.2004, lake margin, under drift material (3, RWC); W of Jemseg near Jemseg River, 45.8255°N, 66.1174°W, 1.VII.2008, R. P. Webster, seasonally flooded marsh, treading vegetation on margin of pool 1, NBM); Grand Lake Meadows P.N.A., 45.8227°N, 66.1209°W, 14-19.V.2010, R. Webster & C. MacKay, old silver maple forest with green ash and seasonally flooded marsh, Lindgren funnel trap (1, AFC); same locality data and forest type, 5-19.VII.2011, 19.VII-5.VIII.2011, M. Roy & V. Webster, Lindgren funnel traps (2, AFC, NBM). **Sunbury Co.**, Maugerville, Portobello Creek N.W.A., 45.8992°N, 66.4248°W, 27.V.2004, 5.VI.2004, R. P. Webster, silver maple forest, margin of small (vernal) pond, in leaf litter (5, RWC); Sheffield, Portobello Creek N.W.A., 45.8952°N, 66.2728°W, 24.VI.2004, R. P. Webster, seasonally flood marsh, treading marsh vegetation (1, RWC). **York Co.**, Douglas, near Nashwaaksis River, 45.9845°N, 66.6908°W, 4.VI.2003, R. P. Webster, silver maple forest, margin of small pond in leaf litter (1, RWC); Charters Settlement, 45.8340°N, 66.7450°W, 27.IV.2006, R. P. Webster, mixed forest, margin of vernal pond in moist leaves (1 ♀, NBM).

**Map 11.**
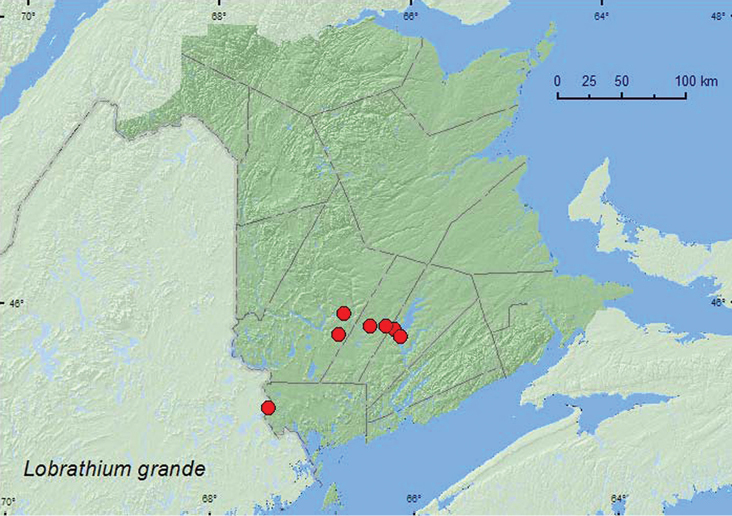
Collection localities in New Brunswick, Canada of *Lobrathium grande*.

##### Collection and habitat data.

Watrous (1981) reported *Lobrathium grande* from a variety of riparian habitats. Adults occurred in moss roots, in leaf litter and sticks at a stream margin, in litter in a North American beaver (*Castor canadensis* Kuhl) lodge and in leaf litter at the margin of a swamp. Larvae were described by Watrous (1981). In New Brunswick, was adults were found in silver maple swamps, seasonally flooded marshes, a mixed forest, and beaver pond margins. Adults were usually found among moist leaves along pond and vernal pond margins or by treading vegetation in marshes. One individual was captured in a Lindgren funnel trap deployed in an old silver maple swamp. Adults were captured during April, May, June, July, and August.

##### Distribution in Canada and Alaska.

ON, QC, **NB**, NS (Campbell and Davies 1991).

#### Lobrathium (Lobrathium) collare

(Erichson, 1840)

http://species-id.net/wiki/Lobrathium_collare

Map 12

##### Material examined.

**New Brunswick, Carleton Co.**, Meduxnekeag Valley Nature Preserve, 46.1931°N, 67.6825°W, 31.V.2005, M.-A. Giguère & R. Webster, mixed forest, river margin, under drift material (1, RWC). **Queens Co.**, Scotchtown near Indian Point (at Grand Lake), 45.8762°N, 66.1816°W, R. P. Webster, 5.VI.2004, lake margin, under drift material (1, RWC); Bayard at Nerepis River, 45.4426°N, 66.3380°W, 30.V.2008, R. P. Webster, river margin, on sand bar in moist sand, collected by lightly splashing sand with water (1, RWC). **Sunbury Co.**, Sheffield, Portobello Creek N.W.A., 45.8952°N, 66.2728°W, 18.VI.2004, R. P. Webster, silver maple forest (swamp), black light trap (1, RWC). **York Co.**, Charters Settlement, 45.8395°N, 66.7391°W, 26.VI.2003, 1.VIII.2004, 10.VI.2005, 29.VI.2005, R. P. Webster, mixed forest, u.v. light (6, RWC).

**Map 12.**
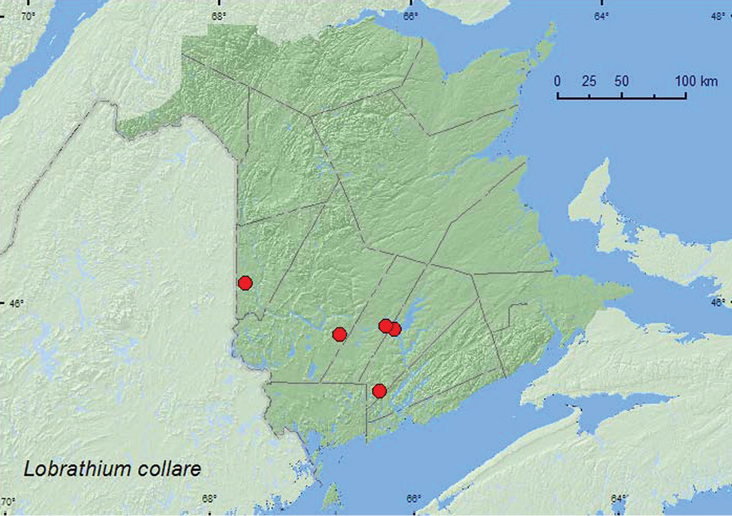
Collection localities in New Brunswick, Canada of *Lobrathium collare*.

##### Collection and habitat data.

Two adults of this species were collected along river margins from under drift material and in sand (splashing), and at an ultraviolet light in a silver maple swamp, and near a mixed forest. Adults were collected during May, June, and August.

##### Distribution in Canada and Alaska.

MB, ON, QC, **NB**, NS (Campbell and Davies 1991).

### Subtribe Medonina Casey, 1905

#### Achenomorphus corticinus

(Gravenhorst, 1802)**

http://species-id.net/wiki/Achenomorphus_corticinus

Map 13

##### Material examined.

**New Brunswick, Sunbury Co.**, Maugerville, Portobello Creek N.W.A., 45.8992°N, 66.4248°W, 18.VI.2004, R. P. Webster, silver maple forest (swamp), black light trap (2 ♀, RWC). **York Co.**, Fredericton, Odell Park, 45.9570°N, 66.6695°W, 19.VI.2005, R. P. Webster, compost (with) wood chips and decaying plant material (2 ♂, 7 ♀, RWC); Charters Settlement, 45.8395°N, 66.7391°W, 17.VII.2004, 9.VII.2008, R. P. Webster, mixed forest, u.v. light (2 ♀, RWC); same locality and collector but, 45.8456°N, 66.7267°W, 10.VI.2010, beaver dam, among sticks and debris near outflow area of dam (1 sex undetermined, RWC).

**Map 13.**
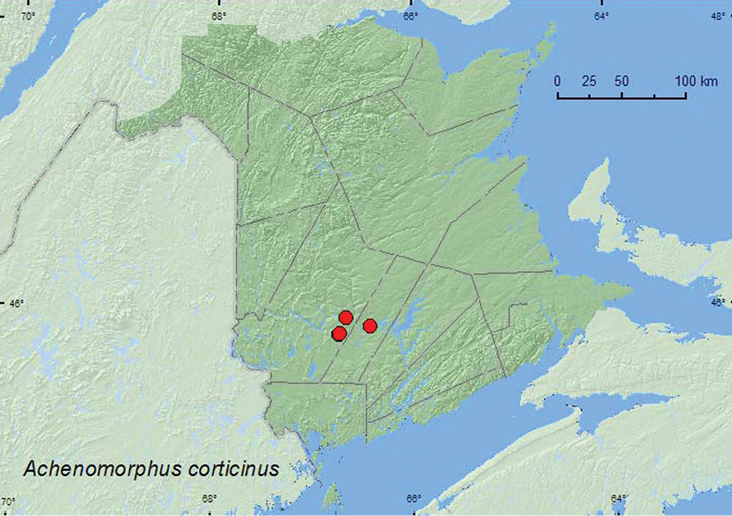
Collection localities in New Brunswick, Canada of *Achenomorphus corticinus*.

##### Collection and habitat data.

In New Brunswick, *Achenomorphus corticinus*adults were collected at ultraviolet light near a mixed forest and in a silver maple swamp. Adults were common in compost with wood chips and decaying plant material. One individual was collected from among sticks and debris near the outflow area of a beaver dam.

##### Distribution in Canada and Alaska.

MB, ON, QC, **NB** (Campbell and Davies 1991).

### Subtribe Stilicina Casey, 1905

#### Rugilus angustatus

(Geoffroy, 1785)

http://species-id.net/wiki/Rugilus_angustatus

Map 14

##### Material examined.

**New Brunswick, Carleton Co.**, Jackson Falls, Bell Forest, 46.2152°N, 67.7190°W, 1.VI.2005, M.-A. Giguère & R. Webster, upper river margin, collected with aerial net between 16:00 and 18:00 h (2 ♀, RWC); **York Co.**, Canterbury, 45.8920°N, 67.6592°W, 8.VI.2004, D. Sabine & R. Webster, hardwood forest, wood pile, under bark (1 ♀, NBM); Charters Settlement, 45.8340°N, 66.7450°W, 27.IV.2005, 30.IV.2005, R. P. Webster, mixed forest, in wood pile, under (loose) bark of spruce (5 ♂, 2 ♀, RWC); Fredericton, at Saint John River, 45.9588°N, 66.6254°W, 7.VI.2005, R. P. Webster, river margin in flood debris (1 ♀, NBM); Fredericton, Odell Park, 45.9570°N, 66.6695°W, 19.VI.2005, R. P. Webster, compost (with) wood chips and decaying plant material (1 ♂, NBM); Fredericton, 45.9361°N, 66.6747°W, 17.VIII.2009, R. P. Webster, beaver dam, outer margin under over-hanging sticks near water (1 ♂, RWC).

**Map 14.**
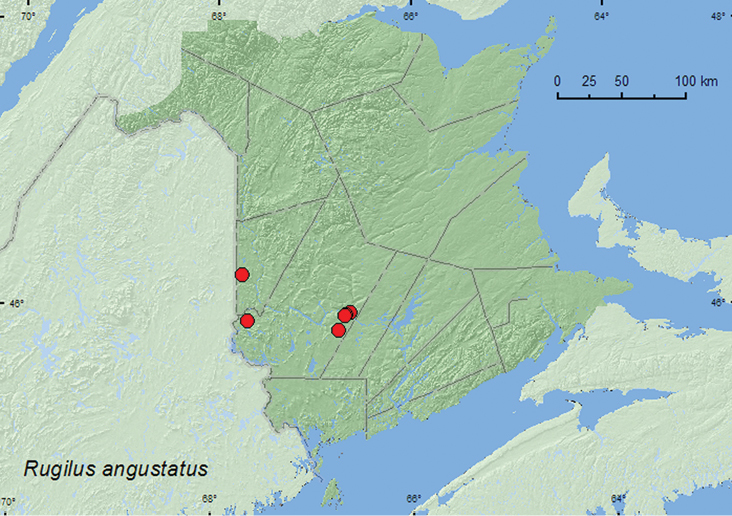
Collection localities in New Brunswick, Canada of *Rugilus angustatus*.

##### Collection and habitat data.

In the Palaearctic region, *Rugilus angustatus* occurs under decaying organic matter along forest borders and watercourses and in meadows (Hoebeke 1995). In New Brunswick, adults of this adventive species were found along river margins, in hardwood and mixed forests, and in a beaver dam. Adults were found under loose bark in wood piles, among composted wood chips and decaying plant material, under overhanging sticks on the outer margin of a beaver dam, and in flood debris on a river margin. Two individuals were collected with an aerial net during evening flight (16:00–18:00 h) on a river margin. Adults were collected during April, June, and August.

##### Distribution in Canada and Alaska.

ON, QC, **NB**, NS (Campbell and Davies 1991; Hoebeke 1995).

#### Rugilus rufipes

Germar, 1836**

http://species-id.net/wiki/Rugilus_rufipes

Map 15

##### Material examined.

**New Brunswick, Carleton Co.**, Jackson Falls, Bell Forest, 46.2152°N, 67.7190°W, 15.IX.2004, R. P. Webster, upper river margin, under litter on clay soil (2 ♂, 1 ♀, NBM, RWC); same locality and collector but 46.2246°N, 67.7206°W, 12.IV.2007, upper river margin, in drift material in area without snow cover, adults very active (2 ♂, 3 ♀, RWC); same locality but 46.2200°N, 67.7231°W, 20-26.V.2009, M.-A. Giguère & R. Webster, mature hardwood forest, Lindgren funnel traps (2, AFC); Meduxnekeag River Valley Nature Preserve, 46.1907°N, 67.6740°W, 20.VI.2006, R. P. Webster, mixed forest, in decaying gilled mushroom, (2 ♂, RWC); Jackson Falls, 46.2257°N, 67.7437°W, 18.VI.2010, R. P. Webster, water falls, splashing moss on rocks near fast flowing water (1, RWC). **York Co.**, Charters Settlement, 45.8395°N, 66.7391°W, 23.IX.2009, R. P. Webster, mixed forest, in decaying (moldy) corncobs and cornhusks (1, RWC).

**Map 15.**
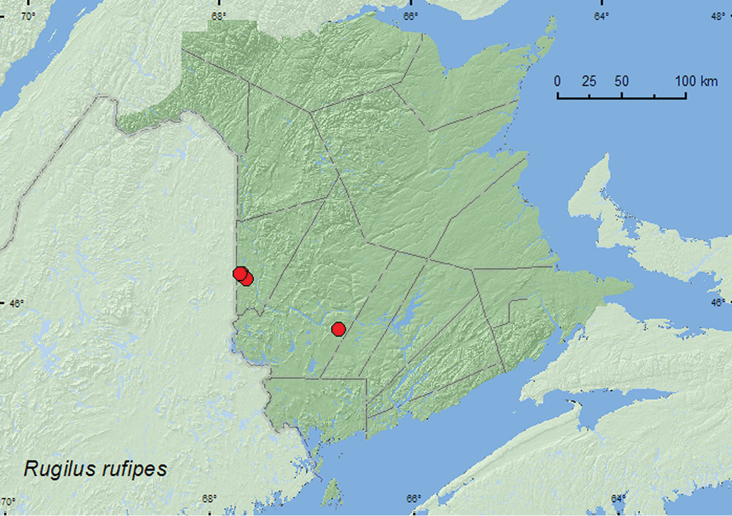
Collection localities in New Brunswick, Canada of *Rugilus rufipes*.

##### Collection and habitat data.

This adventive Palaearctic species lives in both dry and wet habitats in the Palaearctic region, including meadows, fields, heaths, forests, and hilly steppe (Hoebeke 1995). Adults occurred in decaying organic matter and compost, under stones, and among leaves. In New Brunswick, adults were collected from grass litter and drift material along river margins and from decaying gilled mushrooms and decaying (moldy) corncobs and cornhusks in mixed and hardwood forests. One individual was collected from wet moss on rocks adjacent to a waterfall. Adults become active very early in the season when a deep snow cover is still present, as a number of very active adults were collected from a sun-exposed bare patch of drift material on an upper river margin on 12 April when a 60-cm snow pack was still present. Adults were captured during April, May, June, and September.

##### Distribution in Canada and Alaska.

ON, QC, **NB**, (Campbell and Davies 1991; Hoebeke 1995).

### Subtribe Cryptobiina Casey, 1905

#### Homaeotarsus (Gastrolobium) bicolor

(Gravenhorst, 1802)**

http://species-id.net/wiki/Homaeotarsus_bicolor

Map 16

##### Material examined. 

**New Brunswick, Carleton Co**., Belleville, Meduxnekeag River Valley Nature Preserve, 46.1944°N, 67.6832°W, 2.VI.2008, R. P. Webster, river margin, under cobblestone in sand/gravel among scattered grasses (1, RWC).

**Map 16.**
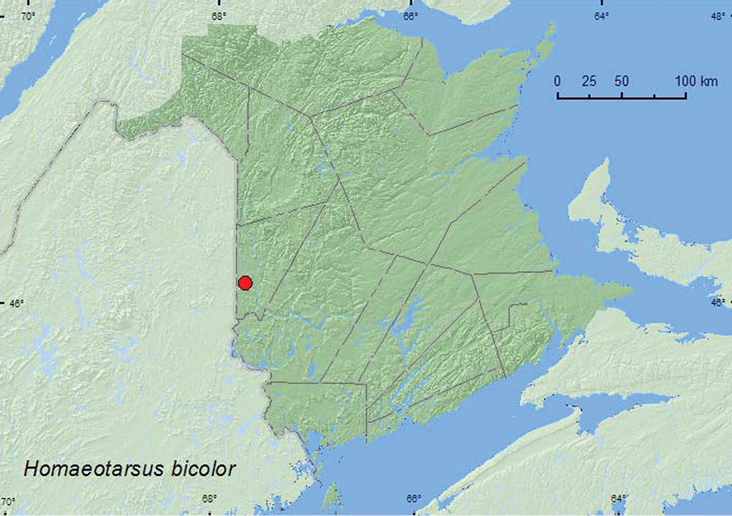
Collection localities in New Brunswick, Canada of *Homaeotarsus bicolor*.

##### Collection and habitat data.

*Homaeotarus* are generally riparian and occur along river margins (Brunke et al. 2011). The single adult of *Homaeotarsus bicolor* from New Brunswick was collected from under a cobblestone along a river margin during early June.

##### Distribution in Canada and Alaska.

ON, QC, NB (Campbell and Davies 1991).

#### Homaeotarsus (Hesperobium) cinctus

(Say, 1830)

http://species-id.net/wiki/Homaeotarsus_cinctus

Map 17

##### Material examined.

**New Brunswick, Carleton Co.**, “Two Mile Brook Fen”, 46.3619°N, 67.6730°W, 6.V.2005, M.-A. Giguère & R. Webster, calcareous cedar fen, open area with sedges, in sphagnum hummock (3, RWC). **Charlotte Co.**, near New River, 45.1616°N, 66.6649°W, 7.VII.2006, R. P. Webster, mixed forest in sedge marsh, treading sedges (1, NBM). **Madawaska Co.**, Loon Lake, 236 m elev., 47.7839°N, 68.3943°W, 21.VI.2010, R. P. Webster, boreal forest, small lake surrounded by sedges, treading sedges and grasses into water (1, NBM). **Restigouche Co.**, Jacquet River Gorge P.N.A., 47.8207°N, 65.9955°W, 12.VIII.2010, R. P. Webster, black spruce bog, treading vegetation (*Carex* & sphagnum) (1, NBM). **Saint John Co.**, Chance Harbour off Rt. 790, 45.1374°N, 66.3633°W, 25.VI.2010, R. P. Webster, saturated green sphagnum mat, treading (1, NBM). **York Co**., Canterbury, Browns Mountain Fen, 45.8967°N, 67.6344°W, 21.VII.2004, D. Sabine, R. Webster, & J. Edsall, calcareous cedar fen, in moss and sphagnum among scattered sedges (1, RWC); same locality and habitat data, 2.V.2005, M.-A. Giguère & R. Webster, open area with sedges, in sphagnum hummock (3, RWC); Charters Settlement, 45.8267°N, 66.7343°W, 14.V.2005, 23.V.2005, R. P. Webster, margin of *Carex* marsh/fen, in sphagnum and leaf litter at base of tree (1 ♂, 2 sex undetermined, RWC); Upper Brockway, 45.5684°N, 67.0993°W, 23.IV.2006, R. P. Webster, forested black spruce bog, in sphagnum (1, NBM); Magundy, 45.8491°N, 67.1573°W, 8.VII.2006, R. P. Webster, kettle hole bog, treading bog margin (1, NBM).

**Map 17.**
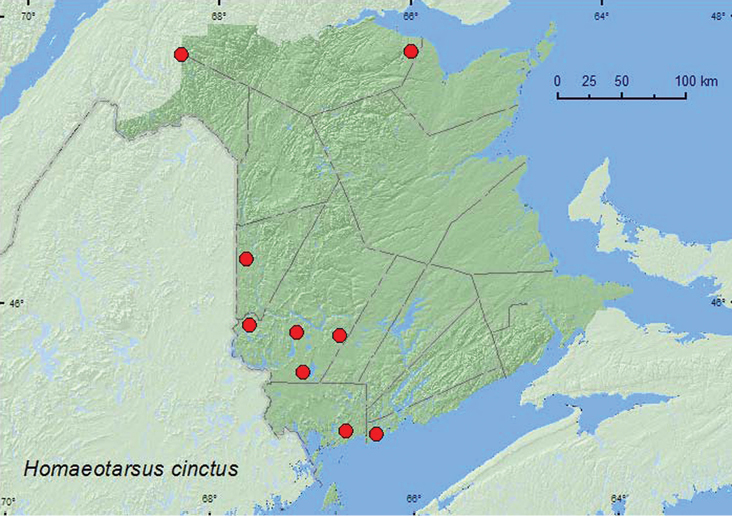
Collection localities in New Brunswick, Canada of *Homaeotarsus cinctus*.

##### Collection and habitat data.

In New Brunswick, *Homaeotarsus cinctus* was found in *Carex* marshes, open calcareous cedar fens, a forested black spruce (*Picea mariana* (Mill.) B.S.P.) bog, and in a kettle hole bog with a floating bog mat. Adults were found in wet to saturated sphagnum with scattered sedges often in a floating mat, among emergent sedges and grasses, and in sphagnum hummocks. Adults were collected by treading these microhabitats. Some adults were sifted from sphagnum and leaf litter at bases of trees on a *Carex* marsh margin. This species was collected during April, May, June, July, and August.

##### Distribution in Canada and Alaska.

BC, AB, ON, QC, **NB**, NS (Campbell and Davies 1991).

#### Homaeotarsus (Hesperobium) cribratus

(LeConte, 1863)**

http://species-id.net/wiki/Homaeotarsus_cribratus

Map 18

##### Material examined.

**New Brunswick, Carleton Co.**, Jackson Falls, Bell Forest, 46.2208°N, 67.7211°W, 19.IV.2005, R. P. Webster, mature hardwood forest, in leaf litter at base of tree (2, RWC); same locality but, 46.2152°N, 67.7190°W, 11.V.2005, M.-A. Giguère & R. Webster, river margin, in drift material (2, RWC); same locality data and collectors, 1.VI.2005, upper river margin, collected with aerial net between 16:00 and 18:00 h (2, RWC); Belleville, Meduxnekeag River Valley Nature Preserve, 46.1888°N, 67.6762°W, 20.V.2005, R. P. Webster, river margin, in flood debris (2, RWC); same locality and collector but, 46.1942°N, 67.6832°W, 2.VI.2008, river margin, under cobblestones (1 ♂, RWC). **York Co**. Fredericton, at Saint John River, 45.9588°N, 66.6254°W, 4.VII.2004, R. P. Webster, river margin, in drift material (mostly maple seeds) (1, RWC).

**Map 18.**
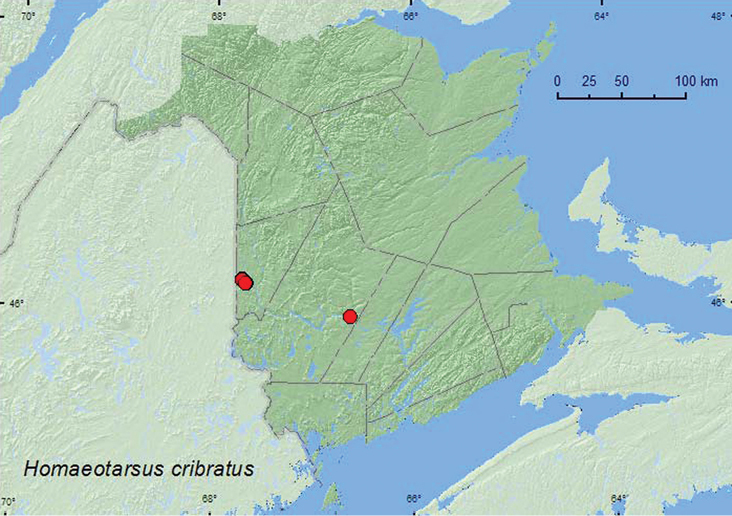
Collection localities in New Brunswick, Canada of *Homaeotarsus cribratus*.

##### Collection and habitat data.

In New Brunswick, most adults of this species were collected along river margins. Adults were collected from flood debris and drift material (maple seeds), and from under a cobblestone. Two adults were collected with an aerial net during an evening flight (16:00–18:00 h) along a river margin, and two individuals were collected from leaf litter at the base of a tree in mature hardwood forest (0.5 km from a river margin) during late April when some snow was still present. This may have been an overwintering site. Adults were collected during April, May, June, and July.

##### Distribution in Canada and Alaska.

ON, QC, **NB** (Campbell and Davies 1991).

#### Homaeotarsus (Hesperobium) pallipes

(Gravenhorst, 1802)**

http://species-id.net/wiki/Homaeotarsus_pallipes

Map 19

##### Material examined.

**New Brunswick, York Co.**, Charters Settlement, 45.8456°N, 66.7267°W, 16.V.2010, R. P. Webster, beaver dam, among sticks and debris near outflow area of dam (1 ♀, RWC).

**Map 19.**
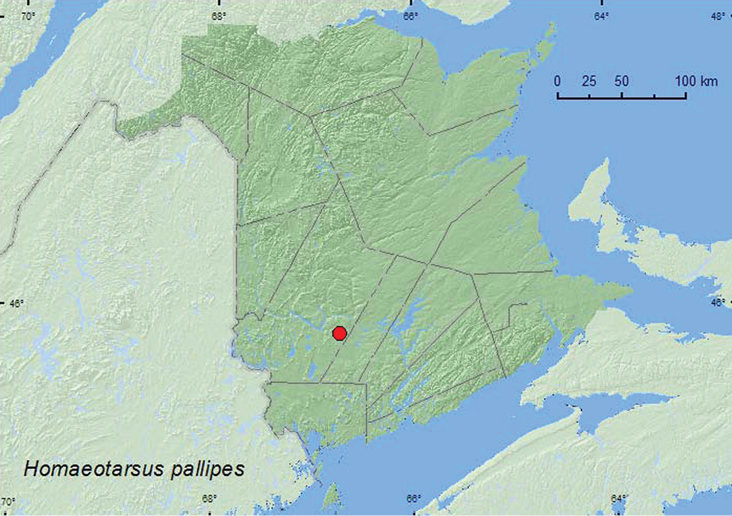
Collection localities in New Brunswick, Canada of *Homaeotarsus pallipes*.

##### Collection and habitat data.

The only specimen from New Brunswick was collected during May from among sticks and debris in a beaver dam near an outflow area with flowing water.

##### Distribution in Canada and Alaska.

ON, QC, **NB** (Campbell and Davies 1991).

## Supplementary Material

XML Treatment for Lathrobium (Lathrobioma) othioides

XML Treatment for Lathrobium (Lathrobium) amplipenne

XML Treatment for Lathrobium (Lathrobium) armatum

XML Treatment for Lathrobium (Lathrobium) confusum

XML Treatment for Lathrobium (Lathrobium) simile

XML Treatment for Lathrobium (Lathrobium) spissicorne

XML Treatment for Lathrobium (Lathrobium) washingtoni

XML Treatment for Lathrobium (Lathrolepta) debile

XML Treatment for Tetartopeus capitosus

XML Treatment for Tetartopeus rubripennis

XML Treatment for Lobrathium (Eulathrobium) grande

XML Treatment for Lobrathium (Lobrathium) collare

XML Treatment for Achenomorphus corticinus

XML Treatment for Rugilus angustatus

XML Treatment for Rugilus rufipes

XML Treatment for Homaeotarsus (Gastrolobium) bicolor

XML Treatment for Homaeotarsus (Hesperobium) cinctus

XML Treatment for Homaeotarsus (Hesperobium) cribratus

XML Treatment for Homaeotarsus (Hesperobium) pallipes
